# Optimizing the robustness of higher-low order coupled networks

**DOI:** 10.1371/journal.pone.0298439

**Published:** 2024-03-14

**Authors:** Chunlin Zheng, Yonglin Hu, Chengjun Zhang, Wenbin Yu, Hui Yao, Yangsong Li, Cheng Fan, Xiaolin Cen

**Affiliations:** 1 School of Computer Science, Nanjing University of Information Science and Technology, Nanjing, China; 2 Jiangsu Second Normal University, Nanjing, China; 3 Information & Computer Center, Tianfeigong Primary School, Nanjing, China; 4 School of Software, Nanjing University of Information Science and Technology, Nanjing, China; 5 Jiangsu Collaborative Innovation Center of Atmospheric Environment and Equipment Technology (CICAEET), Nanjing University of Information Science and Technology, Nanjing, China; 6 Jiangsu Engineering Center of Network Monitoring, Nanjing University of Information Science and Technology, Nanjing, China; AGH University of Science and Technology Faculty of Physics and Applied Computer Science: Akademia Gorniczo-Hutnicza im Stanislawa Staszica w Krakowie Wydzial Fizyki i Informatyki Stosowanej, POLAND

## Abstract

Enhancing the robustness of complex networks is of great practical significance as it ensures the stable operation of infrastructure systems. We measure its robustness by examining the size of the largest connected component of the network after initial attacks. However, traditional research on network robustness enhancement has mainly focused on low-order networks, with little attention given to higher-order networks, particularly higher-low order coupling networks(the largest connected component of the network must exist in both higher-order and low-order networks). To address this issue, this paper proposes robust optimization methods for higher-low order coupled networks based on the greedy algorithm and the simulated annealing algorithm. By comparison, we found that the simulated annealing algorithm performs better. The proposed method optimizes the topology of the low-order network and the higher-order network by randomly reconnecting the edges, thereby enhancing the robustness of the higher-order and low-order coupled network. The experiments were conducted on multiple real networks to evaluate the change in the robustness coefficient before and after network optimization. The results demonstrate that the proposed method can effectively improve the robustness of both low-order and higher-order networks, ultimately enhancing the robustness of higher-low order coupled networks.

## Introduction

As human society continues to rapidly develop, the size and complexity of real-world complex networks are also increasing [1]. The theory of complex networks plays an increasingly prominent role in various fields, such as electricity, education, biology, and the Internet. Malfunctions in these networks can have significant impacts on social life. Therefore, understanding the causes of these malfunctions has become a research focus in network science [2–4]. In recent years, the development of network science has led to the proposal of many network models that have important theoretical value [5–11]. These models have greatly promoted the study of the robustness of complex networks.

The robustness of complex networks is defined as the ability of a network to maintain its structural integrity and functionality in the face of attacks or malfunctions. While individual network robustness has been extensively studied over the past few decades [12–20], many real-world networks are interconnected and interdependent. As technology advances, infrastructure systems are increasingly coupled with the Internet, forming networked physical systems. In power networks, cascading malfunctions between the physical and network systems within power grids have led to widespread power outages. Consequently, many scholars have proposed interdependent network models to describe real-world interdependent systems [21–32].

The aforementioned studies on network robustness primarily focus on low-order networks that are built upon the basic unit of “nodes-edges” and often neglect the higher-order interaction relationships among nodes. With a deeper understanding of real-world networks, it has been discovered that networks contain not only binary interaction relationships but also higher-order interaction relationships where multiple nodes interact simultaneously or in a specific order [33, 34]. For instance, in contemporary educational networks, collaborative relationships frequently arise among multiple individuals simultaneously, such as several students engage in sharing their learning resources with each other to study and grasp common knowledge concepts [35]. Higher-order networks, which are built on network motifs, can better represent the higher-order interaction relationships in networks and are essential for networks to function effectively [36–40]. However, existing studies have revealed that higher-order networks are more fragile than low-order networks and have a strong degree of fragility [41]. Therefore, to ensure the effective protection of networks, it is critical to focus on enhancing the robustness of both higher-order and low-order networks.

The objective of enhancing the robustness of complex networks is to enhance their resilience against diverse interferences and attacks, ensuring network reliability and stability. Currently, there exist various methods for optimizing network robustness, such as edge rearrangement by preserving degrees [42], and building onion-like networks by enhancing loops [43]. The methods for operating edges can be mainly divided into two categories: adding edges and reconnecting edges. Adding edges involves incorporating new connections into the network, including random connection methods, high-betweenness connection methods, and others [44–46], with relatively high optimization costs. In contrast, reconnecting edges aims to improve network robustness by optimizing network topology without altering the number of network edges. This approach typically adjusts the network topology to a highly robust “onion-like” structure, mainly including random edge reconnection methods, intelligent edge reconnection methods, and others [47–49], with high practicality.

This study proposes a robustness optimization approach for higher-order and low-order coupled networks based on a greedy algorithm. The proposed method optimizes the topology of both low-order and higher-order networks using random edge reconnecting, which simultaneously enhances their robustness and ultimately improves the overall robustness of the coupled network. The evaluation metrics of this approach are the relative size of the largest connected component and the robustness coefficient. To verify the effectiveness of this method, experiments are conducted on multiple real network datasets. The results indicate that the proposed method is capable of effectively improving the robustness of both low-order and higher-order networks, and thereby enhancing the overall robustness of the coupled network.

## Related theories

### Higher-order networks and motifs

**Fig 1:**
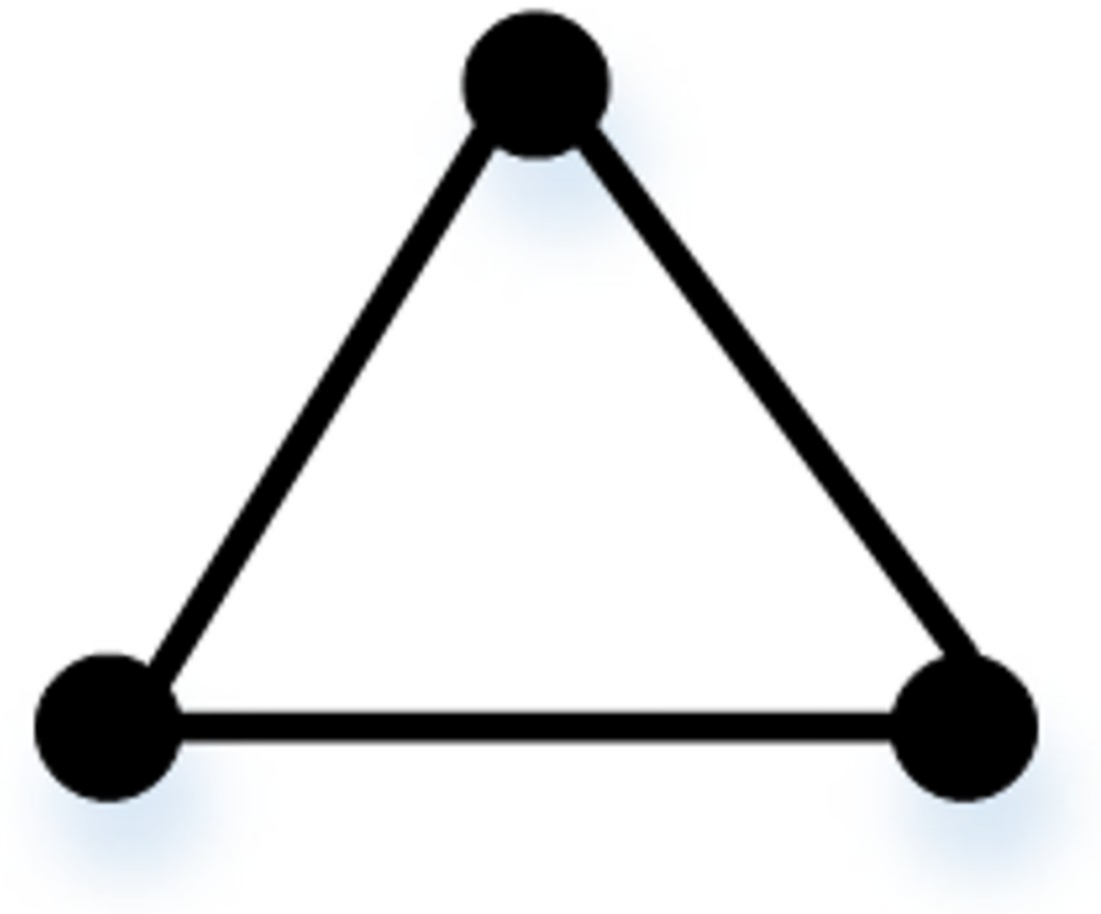
Schematic diagram of a third-order motif.

Real networks are characterized by a significant number of interactive and transitive subgraph structures, also known as network motifs, which serve as the basic building blocks of higher-order network structures [36]. Network motifs play a crucial role in the representation of higher-order networks. Depending on the number of nodes involved, motifs can be categorized as third-order motifs, fourth-order motifs, and so on. Typically, third-order or fourth-order motifs are selected as research units, given that the frequency of higher-order motifs in real networks is relatively low. In undirected networks, the fully connected three-node structure is generally regarded as a third-order motif, as illustrated in Fig 1.

Definition 1: Adjacency matrix based on motifs. For a given motif *M* and a network *G* consisting of *N* nodes, its higher-order adjacency matrix based on *M* can be defined as *W*_*M*_ = {*w*_*ij*_}_*N* × *N*_, where each matrix element *w*_*ij*_ denotes the frequency of appearance of the edge *w*_*ij*_ in the motif M within the network *G*. Mathematically, it can be expressed as:

$$\begin{array}{c} w_{ij}=\sum_{e_{ij}\in M\;\text{,}\;i\neq j}1 \end{array}$$ (1)

Definition 2: Higher-order Network. A higher-order network *G* = (*V*, *E*, *W*_*M*_) is denoted by a set of nodes *V* = {*v*_*i*_∣*i* = 1, 2, 3, …, *n*}, a set of edges *E* = {*e*_*ij*_∣*i*, *j* = 1, 2, 3, …, *m*}, where *e*_*ij*_ is an edge from node *v*_*i*_ to node *v*_*j*_, and the higher-order adjacency matrix *W*_*M*_ is based on the motif *M*.

### Higher-low order coupled networks

**Fig 2:**
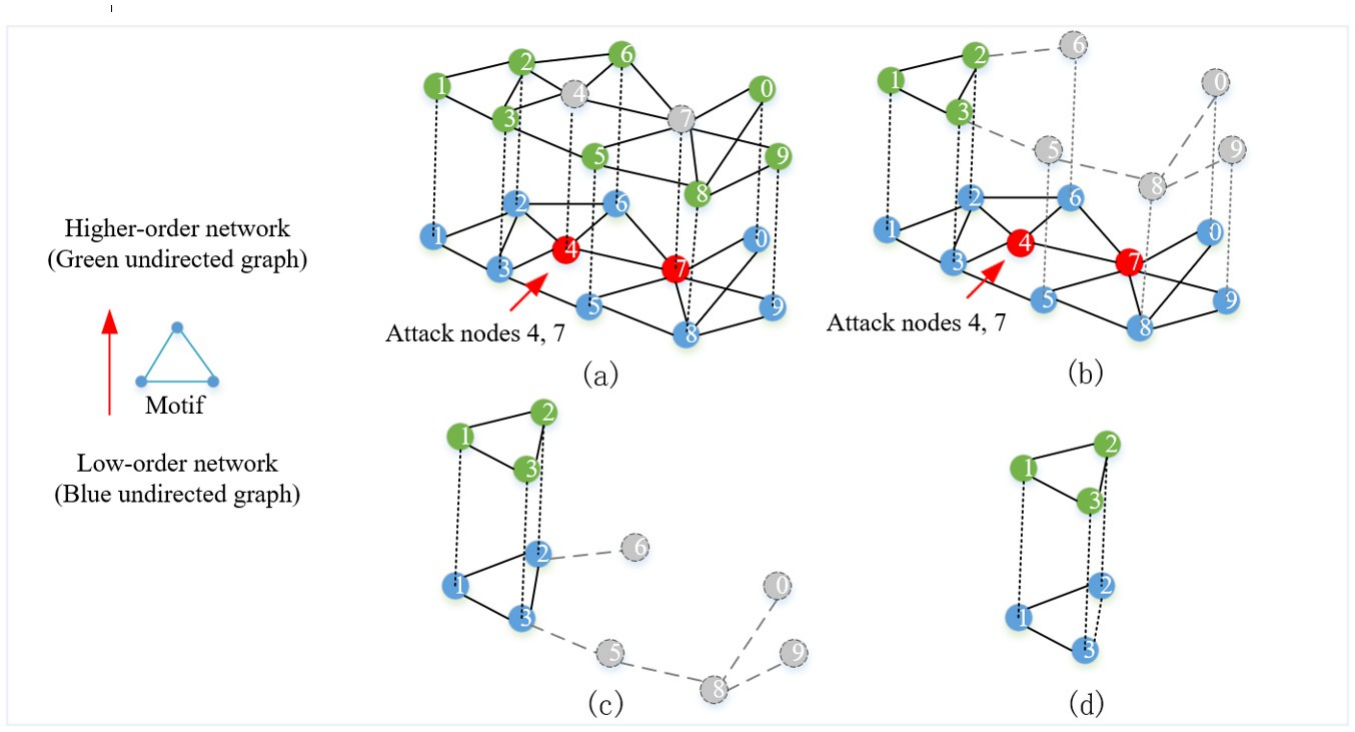
Schematic diagram of higher-low order coupling network and the network after node attacks. The blue network represents the low-order network, and the green network represents the higher-order network built based on the third-order motif derived from the low-order network. (a) shows that after nodes 4 and 7 in the low-order network were attacked, the corresponding nodes 4 and 7 will disappear in the higher-order network; (b) shows that the disappearance of nodes 4 and 7 results in the loss of associated higher-order structure; (c) shows that the disappearance of high-order structure triggers the loss of its corresponding low-order structure; (d) shows the final network state.

Based on the theoretical principles of higher-order networks, a coupling relationship between higher-order and low-order networks exists. Recent research has shown that higher-low order coupled networks are more vulnerable than low-order networks, and the mutual dependence of low-order and higher-order networks can make the robustness of complex networks fragile [50]. The dependence relationship between higher-order and low-order networks can be utilized to construct a higher-low order coupled network model of complex networks, as illustrated in Fig 2. Fig 2 illustrates the network characteristics, where the blue network represents the low-order network, and the green network represents the higher-order network constructed based on the third-order motifs derived from the low-order network. Furthermore, a higher-low order coupled network model is developed to capture the interdependencies between the higher-order and low-order networks. In Fig 2(a), when attacking nodes 4 and 7 in the low-order network, the corresponding nodes 4 and 7 vanish in the higher-order network, leading to the loss of their associated higher-order structures, forming Fig 2(b). Notably, due to the higher-low order coupling, the disappearance of higher-order structures subsequently triggers the loss of their corresponding low-order structures, as shown in Fig 2(c), which eventually leads to the final transition of the network to the structure depicted in Fig 2(d).

In undirected networks, third-order motifs exhibit high robustness and are reflective of the functional characteristics of the network since any pair of nodes in a third-order motif has two shorter independent paths. The failure of a single node has little impact on the connectivity of the third-node motif, as the remaining nodes can still remain connected even if one is removed. Thus, the presence of more third-order motifs in a low-order network indicates higher robustness. However, in higher-order networks, certain nodes have a greater number of third-order motifs, especially nodes with high degree. Moreover, the third-order motif serves as the basic unit of higher-order networks, and the failure of a node can lead to the failure of the third-order motifs to which they belong, ultimately causing other parts of the third-order motifs to fail as well. Consequently, higher-order networks have weaker robustness in the face of intentional attacks.

### Network attack strategies and evaluation metrics

#### Attack strategies

In the present study, a deliberate attack method is employed to assess the node importance based on their degree, and the node with the highest degree in the low-order network along with its connected edges are eliminated during each attack.

#### Evaluation metrics

When a network is subjected to deliberate attacks, the connectivity of the network may be compromised as nodes or edges in the network may fail. The robustness of the network is commonly evaluated by determining the relative size of the largest connected component in the network. Following an attack on a network with *N* nodes, the network may be fragmented into many connected components, with the number of nodes in the largest connected component denoted as *N*′. The relative size of the largest connected component, denoted as *S*, can be calculated as follows:

$$\begin{array}{c} S=\frac{N^{\prime }}{N} \end{array}$$ (2)

The relative size of the largest connected component is a metric that can reveal important information about the structure of a network. For instance, in social networks, it can reflect the extent and effectiveness of information dissemination and communication within the network. A small relative size of the largest connected component indicates the presence of isolated nodes or disconnected components, which may lead to a weak network robustness.

The performance of a network has been evaluated in previous studies using the robustness coefficient [47], which is calculated as follows:

$$\begin{array}{c} R=\frac{1}{N}\sum_{Q=1}^{N}S\left(Q\right) \end{array}$$ (3)

In the formula, *N* denotes the initial number of nodes in the network; *Q* indicates the extent of the attack, with *Q* = *qN* representing the number of nodes that have been removed, where q is the proportion of removed nodes; *S*(*Q*) denotes the relative size of the largest connected component in the network after nodes have been removed; *R* ranges from 1/*N* to 0.5, where a higher value of the network robustness coefficient indicates a stronger network structure and greater resistance to deliberate attacks, and a lower value indicates weaker network robustness.

## Algorithms

The principle of fairness is extremely important in network optimization, as it ensures that the algorithm treats nodes of different levels fairly, rather than just considering some of them. In network optimization, if only some nodes are optimized, it is easy to cause the network to develop abnormally, thus affecting the robustness and reliability of the network. For example, if we only optimize some higher-order nodes and their relationships in a social network, while ignoring individual nodes in the low-order networks, the optimized network structure may seriously affect the stability and robustness of the entire social network. Especially in the case of systemic failures or sudden events, this optimization method is likely to cause network crashes or paralysis. On the contrary, if we follow the principle of fairness, consider nodes of different levels, and comprehensively consider their connections and interactions, we can form a more reasonable, stable, and reliable network structure to deal with various abnormal situations. In any network optimization or improvement algorithm, the principle of fairness should be considered as a basic principle to ensure that the optimized results can effectively support the stability and reliability of the network. This is also an important reason why greedy algorithms can perform well in network optimization.

### Greedy Algorithm 1

This paper proposes a method for optimizing the robustness of higher-order and low-order coupled networks, based on a greedy algorithm. The proposed method aims to improve the robustness of both the low-order and higher-order networks, by adjusting their topology through randomly reconnecting edges, while maintaining their original degree distribution.

This paper proposes a method to optimize the robustness of the low-order network by randomly reconnecting edges. Specifically, the method randomly selects two edges *e*_*ij*_ and *e*_*kl*_ from the low-order network, breaks these edges, and reconnects them to obtain two new edges *e*_*ik*_ and *e*_*jl*_, while ensuring that the new edges do not have self-loops or duplicate edges. The resulting reconnected network is evaluated using the robustness coefficient, and if the coefficient increases, the edge reconnecting process is retained. If the robustness coefficient of the low-order network fails to increase after multiple consecutive edge reconnections, the process of reconnecting edges is stopped to obtain an optimized low-order network. The process is shown in Algorithm 1.

**Algorithm 1 Robustness Optimization Method based on Low-Order Network**

**Input:** The target low-order network *G*, the iteration termination parameter *N*_*max*_

**Output:** The optimized low-order network

1: *N*_*step*_ = 0;

2: **while** *N*_*step*_ < *N*_*max*_ **do**

3:  Calculate *R* of the low-order network before reconnecting the edge;

4:  Randomly selects two edges *e*_*ij*_ and *e*_*kl*_ from the low-order network, breaks these edges and reconnects them to obtain two new edges *e*_*ik*_ and *e*_*jl*_, while ensuring that the new edges do not have self-loops or duplicate edges;

5:  Calculate R* of the low-order network after reconnecting the edges;

6:  **if** R* > *R* **then**

7:   Save the process of reconnecting the edge;

8:   *N*_*step*_ = 0;

9:  **end if**

10:  *N*_*step*_ = *N*_*step*_ + 1;

11: **end while**

12: Return the optimized low-order network;

### Greedy Algorithm 2

Algorithm 1 focuses on optimizing the low-order network by randomly reconnecting edges to increase its robustness coefficient. However, the higher-order network may not see a similar improvement. To address the problem, this paper designs Algorithm 2 to optimize the robustness of the higher-order network. The method first constructs the higher-order network based on the third-order motif of the low-order network, and then calculates its robustness coefficient. The following process is similar to Algorithm 1. Randomly select two connected edges from the low-order network for edge disconnection and reconnection, and calculates the robustness coefficient of the higher-order network after edge reconnection. If the robustness coefficient of the higher-order network increases, the process of reconnecting edges is saved. After multiple consecutive edge disconnections and reconnections, if the robustness coefficient of the higher-order network has not increased, then the edge disconnection and reconnection is stopped, and an optimized low-order network is obtained. The specific steps are outlined in Algorithm 2.

**Algorithm 2 Robustness Optimization of Higher-Order Network based on Greedy Strategy**

**Input:** The target low-order network *G*, the iteration termination parameter *N*_*max*_

**Output:** The optimized low-order network

1: *N*_*step*_ = 0;

2: **while** *N*_*step*_ < *N*_*max*_ **do**

3:  Construct the higher-order network *G*_*high*_ based on the third-order motifs of the low-order network;

4:  Calculate the *R*_*high*_ of the higher-order network before reconnecting edges;

5:  Randomly selects two connected edges *e*_*ij*_ and *e*_*kl*_ from the low-order network and disconnects them, then reconnects them to obtain two new connected edges *e*_*ik*_ and *e*_*jl*_, while ensuring that the new edges do not have self-loops or duplicate edges;

6:  Construct the higher-order network based on the low-order network with the reconnected edges, and calculates the $R_{high}^{*}$ of the higher-order network;

7:  **if** $R_{high}^{*}>R$ **then**

8:   Save the process of rewiring the edge;

9:   *N*_*step*_ = 0;

10:  **end if**

11:  *N*_*step*_ = *N*_*step*_ + 1;

12: **end while**

13: Return the optimized low-order network;

### Greedy Algorithm 3

Algorithm 2 mainly focuses on improving the robustness of higher-order networks, but the robustness of low-order networks may not be improved. Therefore, in order to improve the robustness of low-order network and higher-order network at the same time, Algorithm 3 is designed in this paper. This method first constructs the higher-order network based on the third-order motif of the low-order network, and then randomly selects two connected edges from the low-order network for edge reconnection. The specific process is similar to Algorithm 1. The respective robustness coefficients of the higher-order and low-order networks are calculated after the edge reconnection. If the robustness coefficients of the higher-order network and the low-order network increase at the same time, the process of reconnecting edges is saved. After multiple consecutive edge disconnections and reconnections, the robustness coefficients of the low-order network and the higher-order network cannot be increased at the same time, the edge disconnection reconnection is stopped, and the optimized low-order network is obtained. The specific process is shown in Algorithm 3.

**Algorithm 3 Robustness Optimization Method for Low-Order and Higher-Order Networks Based on Greedy Strategy**

**Input:** The target low-order network *G*, the iteration termination parameter *N*_*max*_

**Output:** The optimized low-order network

1: *N*_*step*_ = 0;

2: **while** *N*_*step*_ < *N*_*max*_ **do**

3:  Construct higher-order network *G*_*high*_ based on third-order motifs of the low-order network;

4:  Calculate the *R*_*low*_ of the low-order network and the *R*_*high*_ of the higher-order network before reconnecting the edge;

5:  Randomly selects two connected edges *e*_*ij*_ and *e*_*kl*_ from the low-order network and disconnects them, then reconnects them to obtain two new connected edges *e*_*ik*_ and *e*_*jl*_, while ensuring that the new edges do not have self-loops or duplicate edges;

6:  Calculate the new $R_{low}^{*}$ of the low-order network after reconnection the edges, and constructs the higher-order network model of the low-order network, and calculates the new $R_{high}^{*}$ of the higher-order network at the same time;

7:  **if** $R_{low}^{*}>R_{low}$ and $R_{high}^{*}>R_{high}$ **then**

8:   Save the process of reconnecting the edge;

9:   *N*_*step*_ = 0;

10:  **end if**

11:  *N*_*step*_ = *N*_*step*_ + 1;

12: **end while**

13: Return the optimized low-order network;

### Simulated Annealing Algorithm

Greedy Algorithm 3 is capable of enhancing the robustness of both the low-order and higher-order networks, although it is susceptible to local optima. To overcome this limitation, we propose the Simulated Annealing Algorithm as a solution. This method initiates by constructing a higher-order network using third-order motifs extracted from the low-order network. Subsequently, two edges are randomly selected from the low-order network and rewired following a process similar to Algorithm 3. The reconnecting process is followed by the computation of robustness coefficients for both the higher-order and low-order networks. If both networks exhibit an increase in their robustness coefficients, the rewiring process is preserved. However, if the robustness coefficients of both networks fail to increase simultaneously, there is a 2% probability (P) of preserving the rewiring. Furthermore, after every 100 iterations, the probability (P) decreases by 1‰. The rewiring process continues iteratively until the robustness coefficients of both the low-order and higher-order networks no longer increase. At this point, the rewiring process is halted, and the optimized low-order network is obtained. The detailed procedure is outlined in Algorithm 4, presented below.

**Algorithm 4 Robustness Optimization Method for Low-Order and Higher-Order Networks Based on Simulated Annealing Strategy**

**Input:** The target low-order network *G*, the iteration termination parameter *N*_*max*_

**Output:** The optimized low-order network

1: *N*_*step*_ = 0;

2: **while** *N*_*step*_ < *N*_*max*_ **do**

3:  Construct higher-order network *G*_*high*_ based on third-order motifs of the low-order network;

4:  Calculate the *R*_*low*_ of the low-order network and the *R*_*high*_ of the higher-order network before reconnecting the edge;

5:  Randomly selects two connected edges *e*_*ij*_ and *e*_*kl*_ from the low-order network and disconnects them, then reconnects them to obtain two new connected edges *e*_*ik*_ and *e*_*jl*_, while ensuring that the new edges do not have self-loops or duplicate edges;

6:  Calculate the new $R_{low}^{*}$ of the low-order network after reconnection the edges, and constructs the higher-order network model of the low-order network, and calculates the new $R_{high}^{*}$ of the higher-order network at the same time;

7:  **if** $R_{low}^{*}>R_{low}$ and $R_{high}^{*}>R_{high}$ **then**

8:   Save the process of reconnecting the edge;

9:   *N*_*step*_ = 0;

10:  **else**

11:   *x* = random.random();

12:   **if** *x* ≤ *p* **then**

13:    Save the process of reconnecting the edge;

14:   **end if**

15:   **if** epoch % 100 == 0 **then**

16:    *p* = *p* − 0.001;

17:   **end if**

18:  **end if**

19:  *N*_*step*_ = *N*_*step*_ + 1;

20: **end while**

21: Return the optimized low-order network;

## Experiments

In order to verify the effectiveness of the algorithm proposed in this paper, the robustness coefficient is used as the evaluation metric to optimize the following networks: the air transportation network Usair, social networks (moreno, wiki-vote, pages-food, rel), email communication network arenas-email, biological networks (Celgans, BCG), and various miscellaneous data networks (G51, ia-infect-dublin, lp-etamacro, lp-agg, model, abb, illc), these datasets are all derived from public network databases [51].

### Experimental datasets

The fundamental characteristics of the network datasets are presented in Table 1, where *N* denotes the overall number of nodes in the network, *E* represents the total number of edges in the network, <*k*> denotes the mean degree of the network, *C* indicates the global clustering coefficient of the network, *r* represents the degree correlation coefficient of the network, and *L* represents the average shortest path length of the network.

**Table 1:** Fundamental characteristics of real-world networks. *N* denotes the overall number of nodes in the network, *E* represents the total number of edges in the network, <*k*> denotes the mean degree of the network, *C* indicates the global clustering coefficient of the network, *r* represents the degree correlation coefficient of the network, and *L* represents the average shortest path length of the network.

Networks	*N*	*E*	<*k*>	*C*	*r*	*L*
ArenasEmail	1133	5451	9.6	0.166	0.0782	3.606
Celgans	453	2025	8.9	0.124	-0.225	2.664
BCG	924	3239	7.011	0.132	-0.179	3.734
G51	1000	5909	11.818	0.14	-0.0681	2.891
Usair	332	2126	12.8	0.396	-0.278	2.738
Moreno	1773	9131	10.3	0.163	-0.0488	3.375
wiki-vote	889	2914	6.5	0.127	-0.0287	4.096
pages-food	620	2102	6.9	0.223	-0.0322	5.089
ia-infect-dublin	410	2765	13.4	0.436	0.225	3.631
lp-etamacro	816	2489	6.1	0.109	-0.279	4.294
lp-agg	612	2407	7.8	0.008	-0.63	3.970
model	798	2991	7.5	0.087	0.0199	3.799
rel	1300	5084	7.8	0.007	-0.730	3.433
abb	313	1553	9.9	0.016	-0.24	3.218
illc	1033	4717	9.1	0.01	-0.329	3.114

### Experimental results analysis

In this section, we investigated the efficacy of algorithms 1, 2, 3 and 4 in optimizing the robustness of both low-order and higher-order networks. Multiple real-world networks were subjected to these three methods for topology optimization. Deliberate attack strategies were employed on both the low-order and higher-order networks to evaluate their robustness, and the relative size S of the largest connected component for each network was recorded. The changes in the relative size of the largest connected component of a network under attack reflect the network’s resilience against such attacks. The robustness coefficient R was also used as a measure of network robustness.

**Fig 3:**
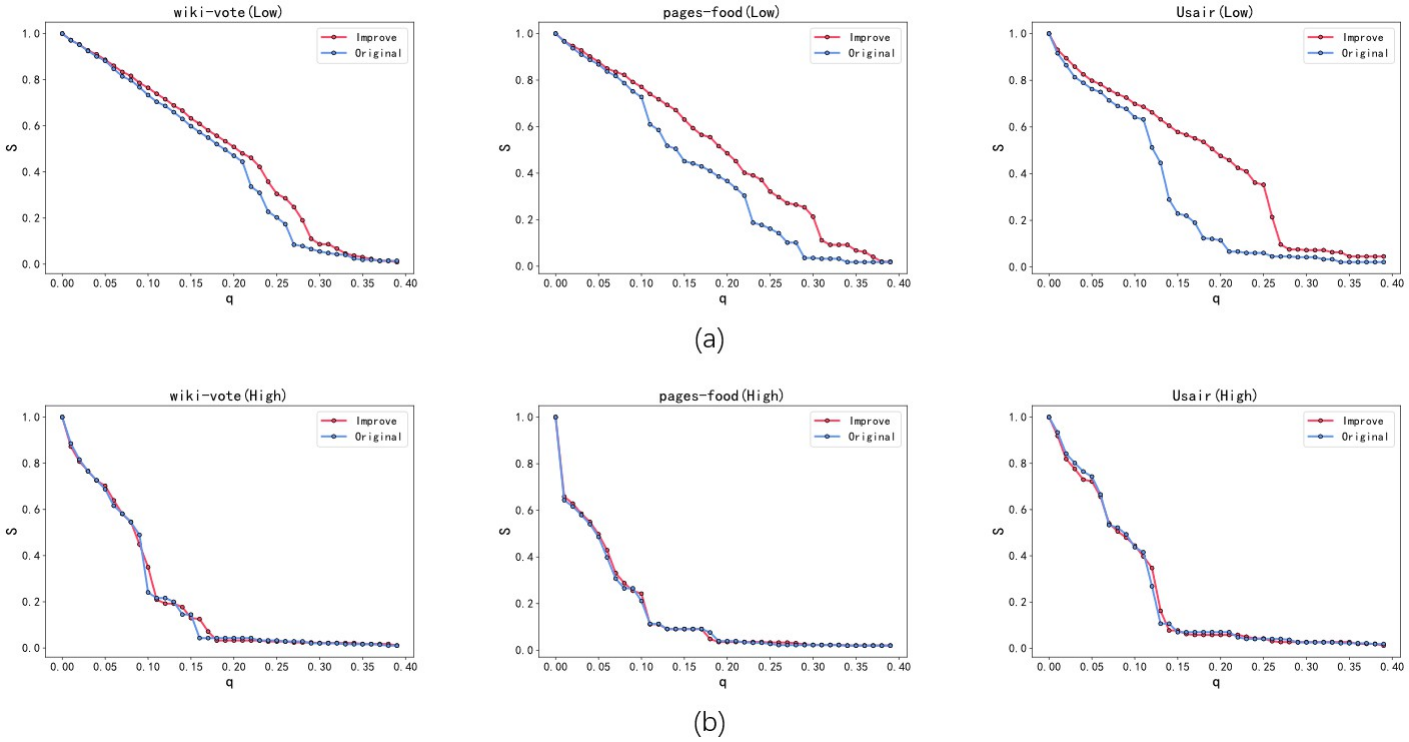
The robustness results of the original network (Original) and its optimized network (Improve) under the optimization of greedy algorithm 1. (a) shows that the S change curve of the low-order network in the optimized network is obviously above the original network, and the low-order network robustness of the optimized network has been significantly improved. (b) shows that the S change curves of the higher-order network in the optimized network and the original network almost overlap, which shows that their high-order networks have similar robustness.

Fig 3 illustrates the experimental results of Algorithm 1. The optimized network (Improve) in the figure has undergone edge reconnection, with approximately 5% of edges being broken and reconnected. The experimental results demonstrate that when the network is subjected to a deliberate attack, the low-order network (Low) and higher-order network (High) of the original network (Original) will rapidly collapse, as manifested by the significant decline in the relative size S of the largest connected component. Specifically, the decline rate of the higher-order network S is more pronounced than that of the low-order network, indicating that the higher-order network is more fragile than the low-order network. As shown in Fig 3(a), after optimization using Algorithm 1, the robustness of the low-order network in the optimized networks was compared with that of the original networks. The S curve of the low-order network in the optimized networks is noticeably above that of the original networks, indicating that the drop rate of S in the low-order network of the optimized networks is slower than that of the original networks. This indicates that Algorithm 1 significantly improved the robustness of the low-order network in the optimized networks. However, as depicted in Fig 3(b), the S curve of the higher-order networks in the optimized and original networks almost overlap, suggesting that their higher-order networks have similar robustness, and that Algorithm 1 can only improve the robustness of the low-order networks without affecting the robustness of the higher-order networks. As a result, a greedy Algorithm 2 was designed in this study to optimize the higher-order networks separately and improve their robustness, given that optimizing only the low-order networks does not enhance the robustness of the higher-order networks.

**Fig 4:**
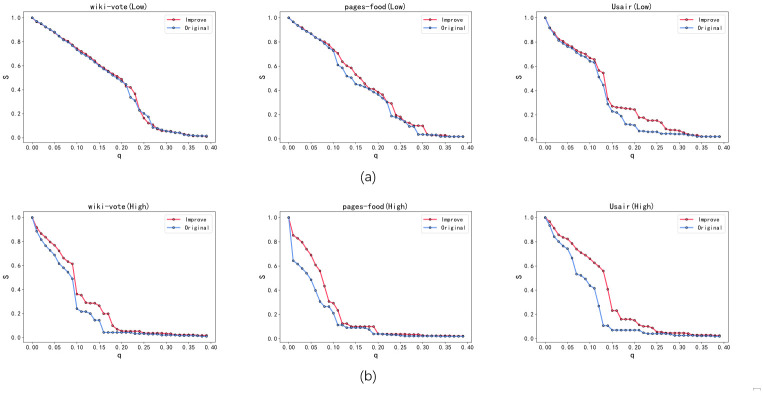
The robustness results of the original network (Original) and its optimized network (Improve) under the optimization of Greedy Algorithm 2. (a) shows the comparison of the robustness of the low-order network in the optimized network and the original network. It can be found that the robustness of the low-order network in the optimized network has been weakly improved. (b) shows that the S change curve of the higher-order network in the optimized network is obviously above the original network, indicating that the robustness of the higher-order network in the optimized network has been significantly improved.

Fig 4 presents the outcomes of Algorithm 2, wherein the optimized networks undergo approximately 5% reconnecting of their edges. As shown in Fig 4(a), the robustness of the low-order networks between the original and optimized networks is compared, indicating that the robustness of the low-order networks in the optimized networks is slightly improved, with the Usair network showing a more significant improvement. However, the optimized wiki-vote network’s low-order network’s robustness is significantly weaker than that of the original network when around 25% of the nodes are attacked, despite a general improvement in its robustness. Fig 4(b) illustrates that the S curve of the higher-order network in the three optimized networks is significantly above that of the original network after optimization with Algorithm 2, suggesting that the optimized network’s S decrease rate is slower, and the higher-order network robustness has been considerably improved. Algorithm 2 solely concentrates on improving the robustness of higher-order networks. leading to a significant improvement in their robustness while also generally enhancing the low-order networks’ robustness. However, in some networks, such as the wiki-vote network demonstrated in Fig 4(a), the optimized low-order networks’ robustness may be weaker than that of the original networks when some nodes are attacked. To address this problem, this study proposes Greedy Algorithm 3, which simultaneously enhances the robustness of both low-order and higher-order networks, resulting in an overall improvement in the network’s robustness.

**Fig 5:**
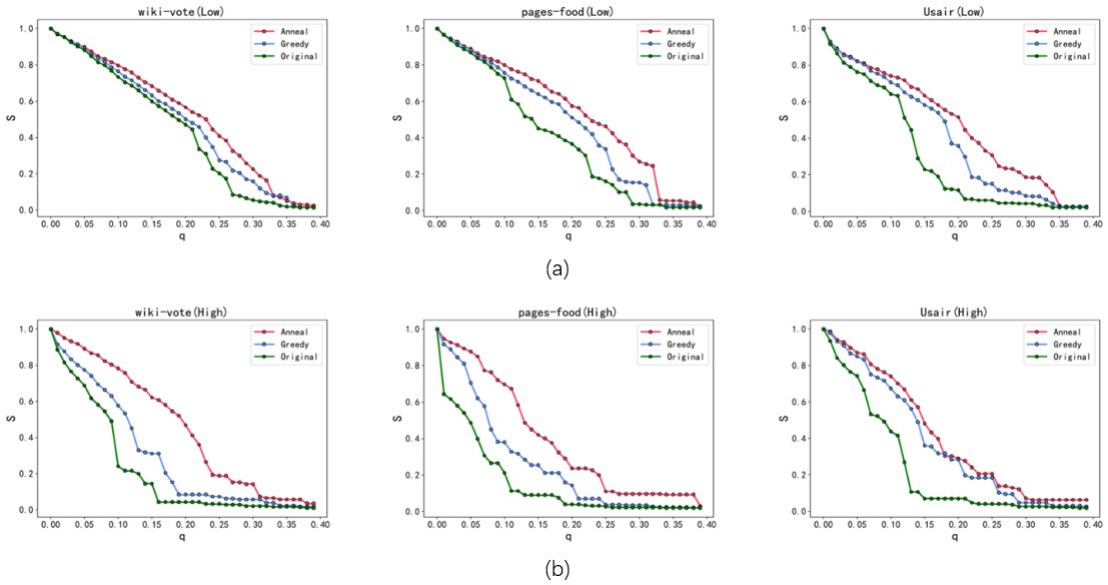
The robustness results of the original network (Original), the network optimized using Greedy Algorithm 3 (Greedy), and the network optimized using Simulated Annealing Algorithm (Anneal). Both (a) and (b) demonstrate the leading advantages of the simulated annealing algorithm, and illustrate that both our proposed greedy algorithm 3 and the simulated annealing algorithm effectively enhance the robustness of low-order networks and higher-order networks.

Fig 5 illustrates the experimental results obtained from the application of Greedy Algorithm 3 and Simulated Annealing Algorithm. The optimized networks depicted in the figure involve reconnecting approximately 5% of the edges. The findings substantiate the effectiveness of our proposed Greedy Algorithm 3 in enhancing the robustness of both the low-order and higher-order networks. Nonetheless, considering the susceptibility of the greedy algorithm to local optima issues, we augmented it with the Simulated Annealing Algorithm, which exhibited significantly superior performance compared to Greedy Algorithm 3. In Fig 5(a), the curve representing the variation of the S metric in the optimized low-order network consistently surpasses that of the original network. Both Greedy Algorithm 3 and Simulated Annealing Algorithm effectively bolster the robustness of the low-order network compared to Greedy Algorithm 2. Notably, the Simulated Annealing Algorithm exhibits a notable advantage in improving the robustness of the low-order network. Fig 5(b) showcases the considerable improvement in the robustness of the higher-order network following optimization with Greedy Algorithm 3 and Simulated Annealing Algorithm. The Simulated Annealing Algorithm consistently maintains a leading advantage. Consequently, both our proposed Greedy Algorithm 3 and Simulated Annealing Algorithm effectively enhance the robustness of both the low-order and higher-order networks, with the Simulated Annealing Algorithm surpassing the performance of Greedy Algorithm 3.

In order to compare the robustness improvement ratios among Greedy Algorithm 2, Greedy Algorithm 3, and Simulated Annealing Algorithm, this paper conducts separate optimizations of partial networks using these algorithms. Firstly, the original network with 1% of the edges are optimized multiple times to obtain the most robust optimized network, and then 1% edges are further optimized on this optimized network to obtain the optimal robustness optimized network. This optimization process is repeated until approximately 10% of the edges in the original network have been optimized. The improvement ratio of robustness is defined as follows:

$$\begin{array}{c} \Delta R=\frac{R^{\prime }-R}{R} \end{array}$$ (4)

**Fig 6:**
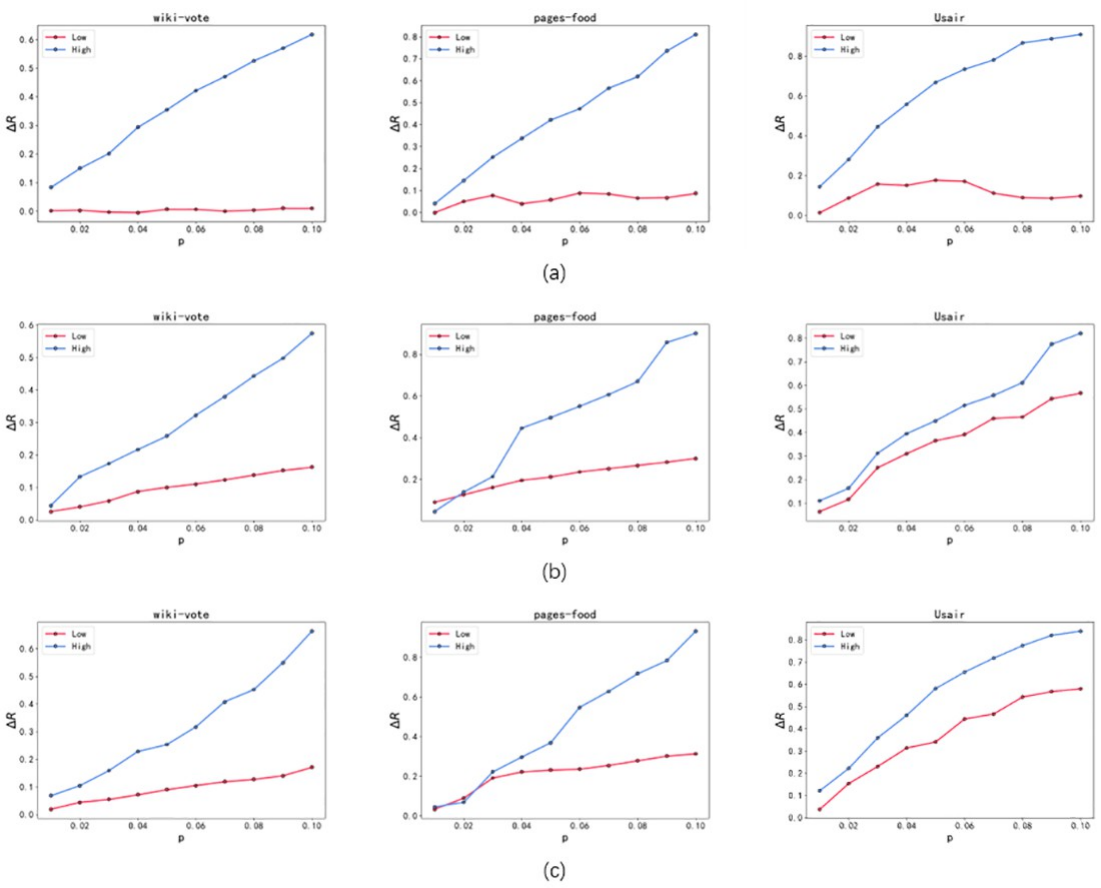
The robustness improvement ratio of low-order network (Low) and higher-order network (High) under the optimization of Greedy Algorithm 2, Greedy Algorithm 3, and Simulated Annealing Algorithm. (a) shows that greedy algorithm 2 has the smallest enhancement effect on network robustness. (b) and (c) illustrate that the greedy algorithm 3 and the simulated annealing algorithm show nearly linear growth in improving network robustness, while the simulated annealing algorithm shows higher optimization efficiency than the greedy algorithm 3.

The parameters *R* and *R*′ represent the robustness coefficients of the original network and the optimized network respectively. The corresponding experimental results are shown in Fig 6, where *p* represents the optimization ratio of the edges.

Based on the findings from Fig 6, all three algorithms, Greedy Algorithm 2, Greedy Algorithm 3 and Simulated Annealing Algorithm, exhibit effective improvements in the robustness of the higher-order network. Throughout the edge optimization process ranging from 1% to 10%, the robustness of the higher-order network in the original network demonstrates a nearly linear growth pattern. Specifically, as depicted in Fig 6(a), Greedy Algorithm 2 demonstrates a significant enhancement in the robustness of the higher-order network, while its impact on the robustness of the low-order network is minimal. Fig 6(b) and 6(c) illustrate that both Greedy Algorithm 3 and Simulated Annealing Algorithm exhibit nearly linear growth in improving the robustness of the low-order network during the optimization process, with the “Usair” network displaying the most prominent effect. In comparison, Greedy Algorithm 3 and Simulated Annealing Algorithm demonstrate higher optimization efficiency than Greedy Algorithm 2. While the visual differences between the Simulated Annealing Algorithm and the Greedy Algorithm 3 in Fig 6 may appear negligible, it is crucial to consider that this metric is associated with the area plot in Fig 5. Hence, even minor disparities in Fig 6 correspond to substantial deviations in the curves presented in Fig 5. However, as the edge optimization proportion increases, it becomes evident that Simulated Annealing Algorithm surpasses Greedy Algorithm 3 in enhancing the original network’s robustness.

Table 2 showcases the ratios of improvement in robustness for both the low-order and higher-order networks following the application of the simulated annealing algorithm for optimization across all datasets. The table displays various edge reconnecting percentages, including 5%, 10%, 20%, and 100%. Upon optimizing with the simulated annealing algorithm, a significant enhancement in the robustness of both the low-order and higher-order networks can be achieved by reconnecting approximately 5% of the edges. However, certain cases reveal a decline in the network’s optimization performance as the reconnecting percentage increases. This phenomenon can be attributed to two factors: random variability and trade-offs. Trade-offs involve making compromises by sacrificing the robustness of either the low-order or higher-order network to improve the other network.

**Table 2:** Robustness improvement ratios of low-order and higher-order networks.

	Low-order Network (5%)	Higher-order Network (5%)	Low-order Network (10%)	Higher-order Network(10%)	Low-order Network (20%)	Higher-order Network (20%)	Low-order Network (100%)	Higher-order Network (100%)
Usair	52%	62%	57%	75%	69%	86%	58%	61%
Moreno	19%	26%	23%	31%	34%	51%	52%	76%
wiki-vote	20%	56%	18%	88%	32%	91%	37%	94%
pages-food	37%	65%	32%	74%	45%	82%	46%	85%
arenas-email	10%	31%	14%	45%	19%	54%	28%	59%
Celgans	40%	16%	39%	17%	90%	41%	73%	53%
BCG	36%	24%	56%	59%	61%	70%	64%	98%
G51	8%	11%	7%	10%	8%	11%	20%	30%
ia-infect-dublin	9%	24%	8%	28%	11%	38%	20%	43%
lp-etamacro	20%	37%	28%	57%	41%	67%	50%	87%
lp-agg	10%	61%	15%	70%	35%	85%	50%	85%
model	10%	71%	14%	78%	26%	80%	28%	86%
rel	6%	56%	9%	51%	12%	%	14%	83%
abb	5%	33%	6%	34%	6%	31%	6%	31%
illc	31%	32%	60%	50%	91%	75%	14%	86%

Table 3 presents the statistical characteristics of both the original and optimized networks for all datasets after the application of the Simulated Annealing Algorithm. In the table, <*k*> represents the average degree of the original network, *C* is the global clustering coefficient of the original network, *r* is the degree correlation coefficient of the original network, *L* is the average shortest path length of the original network, <*k*>′ represents the average degree of the optimized network, *C*′ is the global clustering coefficient of the optimized network, and *r*′ is the degree correlation coefficient of the optimized network, *L*′ is the average shortest path length of the optimized network. Compared to the original network, the global clustering coefficient and the average shortest path length of the optimized network has slightly decreased, and the degree correlation coefficient has slightly increased. This means that the correlation between node degrees in the optimized network is not as strong, and the structure of the optimized network is looser. In this case, the network robustness is enhanced, indicating that the structural characteristics of randomness and dispersion are more conducive to improving the robustness of both low-order and higher-order networks.

**Table 3:** Statistical characteristics of real-world networks before and after optimizing 5% of the edges. Among them, <*k*>, *C*, *r* and *L* represent the network statistical features before optimization, while <*k*>′, *C*′, *r*′ and *L*′ denote the statistical features of the network after optimizing 5% of the edges.

Networks	<*k*>	*C*	*r*	*L*	<*k*>′	*C*′	*r*′	*L*′
ArenasEmail	9.6	0.166	0.0782	3.606	9.6	0.162	0.0959	3.567
Celgans	8.9	0.124	-0.225	2.664	8.9	0.123	-0.220	2.634
BCG	7.011	0.132	-0.179	3.734	7.011	0.129	-0.173	3.702
G51	11.818	0.14	-0.0681	2.891	11.818	0.134	-0.630	2.885
Usair	12.8	0.396	-0.278	2.738	12.8	0.387	-0.207	2.652
Moreno	10.3	0.163	-0.0488	3.375	10.3	0.159	-0.0486	3.257
wiki-vote	6.5	0.127	-0.0287	4.096	6.5	0.124	-0.0288	3.827
pages-food	6.9	0.223	-0.0322	5.089	6.9	0.213	-0.031	3.952
ia-infect-dublin	13.4	0.436	0.225	3.631	13.4	0.427	0.237	3.269
lp-etamacro	6.1	0.109	-0.279	4.294	6.1	0.097	-0.246	4.109
lp-agg	7.8	0.008	-0.63	3.970	7.8	0.007	-0.615	3.710
model	7.5	0.087	0.0199	3.799	7.5	0.086	0.0323	3.734
rel	7.8	0.007	-0.730	3.433	7.8	0.006	-0.7	3.346
abb	9.9	0.016	-0.24	3.218	9.9	0.018	-0.023	3.128
illc	9.1	0.01	-0.329	3.114	9.1	0.013	-0.322	2.941

## Conclusion

This paper proposes a robustness optimization method for higher-order and low-order coupled networks based on a greedy algorithm. The method aims to optimize the robustness of higher-order or low-order networks by randomly reconnecting a small number of edges without changing the degree distribution, thereby improving the topology of the network and ultimately enhancing the robustness of both low-order and higher-order networks. Experimental analysis demonstrates that Greedy Algorithm 1, which targets low-order network robustness, can only improve the robustness of low-order networks. Meanwhile, Greedy Algorithm 2, which optimizes higher-order networks robustness, can only enhance the robustness of higher-order networks. Both Greedy Algorithm 3 and Simulated Annealing Algorithm, targeting the enhancement of robustness in both the low-order and higher-order networks, exhibit effective performance. In comparison to Greedy Algorithm 2 and Greedy Algorithm 3, the optimization performance of Simulated Annealing Algorithm is superior, demonstrating its superiority in improving the robustness of the networks. In this paper, the Simulated Annealing Algorithm was applied to optimize multiple real-world networks, and achieved good optimization results. When the real networks were optimized by 5% of edges, both the low-order and higher-order network robustness were significantly improved. However, the method proposed in this paper is designed for undirected networks, and whether it is applicable to directed networks needs further research in the future. Additionally, in the future, alternative methods such as heuristic methods, methods based on complex network dynamics, and methods utilizing deep learning will be explored for improving the robustness of higher-low order coupled networks.

## Supporting information

S1 Data (ZIP)

## References

[1] Nicol DM, Yan G. High-performance simulation of low-resolution network flows. Simulation. 2006;82(1):21–42. doi: 10.1177/0037549706066093

[2] Werner NE, Bumpus MF, Rock D. Involvement in internet aggression during early adolescence. Journal of youth and adolescence. 2010;39:607–619. doi: 10.1007/s10964-009-9419-7 20422350

[3] Wang J, Xiong W, Wang R, Cai S, Wu D, Wang W, et al. Effects of the information-driven awareness on epidemic spreading on multiplex networks. Chaos: An Interdisciplinary Journal of Nonlinear Science. 2022;32(7):073123. doi: 10.1063/5.0092031 35907734

[4] Cai Q, Alam S, Pratama M, Liu J. Robustness evaluation of multipartite complex networks based on percolation theory. IEEE Transactions on Systems, Man, and Cybernetics: Systems. 2020;51(10):6244–6257. doi: 10.1109/TSMC.2019.2960156

[5] Erdős P, Rényi A. On random graphs I. Publicationes Mathematicae (Debrecen); 1959.

[6] Erdős P, Rényi A, et al. On the evolution of random graphs. Publ Math Inst Hung Acad Sci. 1960;5(1):17–60.

[7] Price DJDS. Networks of scientific papers: The pattern of bibliographic references indicates the nature of the scientific research front. Science. 1965;149(3683):510–515. doi: 10.1126/science.149.3683.510 14325149

[8] Zhu L, Basu S, Jarrow RA, Wells MT. High-Dimensional Estimation, Basis Assets, and the Adaptive Multi-Factor Model. The Quarterly Journal of Finance. 2020;10(04):2050017. doi: 10.1142/S2010139220500172

[9] Price DdS. A general theory of bibliometric and other cumulative advantage processes. Journal of the American society for Information science. 1976;27(5):292–306. doi: 10.1002/asi.4630270505

[10] Watts DJ, Strogatz SH. Collective dynamics of ‘small-world’networks. nature. 1998;393(6684):440–442. doi: 10.1038/30918 9623998

[11] Barabási AL, Albert R. Emergence of scaling in random networks. science. 1999;286(5439):509–512. doi: 10.1126/science.286.5439.509 10521342

[12] Albert R, Jeong H, Barabási AL. Error and attack tolerance of complex networks. nature. 2000;406(6794):378–382. doi: 10.1038/35019019 10935628

[13] Cohen R, Erez K, Ben-Avraham D, Havlin S. Resilience of the internet to random breakdowns. Physical review letters. 2000;85(21):4626. doi: 10.1103/PhysRevLett.85.4626 11082612

[14] Cohen R, Erez K, Ben-Avraham D, Havlin S. Breakdown of the internet under intentional attack. Physical review letters. 2001;86(16):3682. doi: 10.1103/PhysRevLett.86.3682 11328053

[15] Yang B, Huang X, Hu X, Cheng W, Pei Z, Li X. Optimizing Robustness of Core-Periphery Structure in Complex Networks. IEEE Transactions on Circuits and Systems II: Express Briefs. 2021;68(12):3572–3576.

[16] Frutos Bernal E, Martín del Rey A. Study of the structural and robustness characteristics of madrid metro network. Sustainability. 2019;11(12):3486. doi: 10.3390/su11123486

[17] Lou Y, Wu R, Li J, Wang L, Chen G. A convolutional neural network approach to predicting network connectedness robustness. IEEE Transactions on Network Science and Engineering. 2021;8(4):3209–3219. doi: 10.1109/TNSE.2021.3107186

[18] Ratnayake P, Weragoda S, Wansapura J, Kasthurirathna D, Piraveenan M. Quantifying the robustness of complex networks with heterogeneous nodes. Mathematics. 2021;9(21):2769. doi: 10.3390/math9212769

[19] Markina-Khusid A, Jacobs RB, Antul L, Cho L, Tran HT. A complex network framework for validated assessments of systems of systems robustness. IEEE Systems Journal. 2021;16(1):1092–1102. doi: 10.1109/JSYST.2021.3064817

[20] Yang YY, Feng B, Zhang L, Xue SH, Xie XL, Wang JR. Robustness measurement of scale-free networks based on motif entropy. Chinese Physics B. 2022;31(8):080201. doi: 10.1088/1674-1056/ac6942

[21] Buldyrev SV, Parshani R, Paul G, Stanley HE, Havlin S. Catastrophic cascade of failures in interdependent networks. Nature. 2010;464(7291):1025–1028. doi: 10.1038/nature08932 20393559

[22] Parshani R, Buldyrev SV, Havlin S. Interdependent networks: Reducing the coupling strength leads to a change from a first to second order percolation transition. Physical review letters. 2010;105(4):048701. doi: 10.1103/PhysRevLett.105.048701 20867893

[23] Parshani R, Buldyrev SV, Havlin S. Critical effect of dependency groups on the function of networks. Proceedings of the National Academy of Sciences. 2011;108(3):1007–1010. doi: 10.1073/pnas.1008404108 21191103 PMC3024657

[24] Peng H, Xie Z, Zhao D, Zhong M, Han J, Wang W, et al. Reliability analysis of interdependent hypergraph network under different attack strategies. International Journal of Modern Physics C (IJMPC). 2023;34(02):1–20.

[25] Sun S, Wu Y, Ma Y, Wang L, Gao Z, Xia C. Impact of degree heterogeneity on attack vulnerability of interdependent networks. Scientific reports. 2016;6(1):32983. doi: 10.1038/srep32983 27609483 PMC5016735

[26] Ermagun A, Tajik N. Recovery patterns and physics of the network. PloS one. 2021;16(1):e0245396. doi: 10.1371/journal.pone.0245396 33465154 PMC7815135

[27] Smolyak A, Levy O, Vodenska I, Buldyrev S, Havlin S. Mitigation of cascading failures in complex networks. Scientific reports. 2020;10(1):16124. doi: 10.1038/s41598-020-72771-4 32999338 PMC7528121

[28] Turalska M, Swami A. Greedy control of cascading failures in interdependent networks. Scientific reports. 2021;11(1):3276. doi: 10.1038/s41598-021-82843-8 33558578 PMC7870659

[29] Zheng K, Liu Y, Gong J, Wang W. Robustness of circularly interdependent networks. Chaos, Solitons & Fractals. 2022;157:111934. doi: 10.1016/j.chaos.2022.111934

[30] Ding L, Xie L, Xu XK. Rich-club impact on cascading failures in interdependent power and communication networks. IEEE Journal on Emerging and Selected Topics in Circuits and Systems. 2022;12(1):115–123. doi: 10.1109/JETCAS.2022.3151413

[31] Gao Y, Liu J, He H, Zhou J, Chen S. Multiple phase transitions in ER edge-coupled interdependent networks. New Journal of Physics. 2022;24(2):023023. doi: 10.1088/1367-2630/ac5055

[32] Wang J, Cai SM, Zhou T. Immunization of cooperative spreading dynamics on complex networks. Complexity. 2021;2021:1–7. doi: 10.1155/2021/9650741

[33] Bianconi G. Higher-order networks. Cambridge University Press; 2021.

[34] Liao H, Liu QX, Huang ZC, Lu KZ, Yeung CH, Zhang YC. Accumulative Time Based Ranking Method to Reputation Evaluation in Information Networks. Journal of Computer Science and Technology. 2022;37(4):960–974. doi: 10.1007/s11390-021-0471-4

[35] Choi SY, Kang JM. An adaptive system supporting collaborative learning based on a location-based social network and semantic user modeling. International Journal of Distributed Sensor Networks. 2012;8(3):506810. doi: 10.1155/2012/506810

[36] Benson AR, Gleich DF, Leskovec J. Higher-order organization of complex networks. Science. 2016;353(6295):163–166. doi: 10.1126/science.aad9029 27387949 PMC5133458

[37] Li X, Zhao C, Hu Z, Yu C, Duan X. Revealing the character of journals in higher-order citation networks. Scientometrics. 2022;127(11):6315–6338. doi: 10.1007/s11192-022-04518-z

[38] Baccini F, Geraci F, Bianconi G. Weighted simplicial complexes and their representation power of higher-order network data and topology. Physical Review E. 2022;106(3):034319. doi: 10.1103/PhysRevE.106.034319 36266916

[39] Sun H, Bianconi G. Higher-order percolation processes on multiplex hypergraphs. Physical Review E. 2021;104(3):034306. doi: 10.1103/PhysRevE.104.034306 34654130

[40] Majhi S, Perc M, Ghosh D. Dynamics on higher-order networks: A review. Journal of the Royal Society Interface. 2022;19(188):20220043. doi: 10.1098/rsif.2022.0043 35317647 PMC8941407

[41] Xia D, Li Q, Lei Y, Shen X, Qian M, Zhang C. Extreme vulnerability of high-order organization in complex networks. Physics Letters A. 2022;424:127829. doi: 10.1016/j.physleta.2021.127829

[42] Chan H, Akoglu L. Optimizing network robustness by edge rewiring: a general framework. Data Mining and Knowledge Discovery. 2016;30:1395–1425. doi: 10.1007/s10618-015-0447-5

[43] Chujyo M, Hayashi Y. A loop enhancement strategy for network robustness. Applied Network Science. 2021;6(1):1–13. doi: 10.1007/s41109-020-00343-6

[44] Cao XB, Hong C, Du WB, Zhang J. Improving the network robustness against cascading failures by adding links. Chaos, Solitons & Fractals. 2013;57:35–40. doi: 10.1016/j.chaos.2013.08.007

[45] Chen CY, Zhao Y, Qin H, Meng X, Gao J. Robustness of interdependent scale-free networks based on link addition strategies. Physica A: Statistical Mechanics and its Applications. 2022;604:127851. doi: 10.1016/j.physa.2022.127851

[46] Lonapalawong S, Yan J, Li J, Ye D, Chen W, Tang Y, et al. Reducing power grid cascading failure propagation by minimizing algebraic connectivity in edge addition. Frontiers of Information Technology & Electronic Engineering. 2022;23(3):382–397. doi: 10.1631/FITEE.2000596

[47] Schneider CM, Moreira AA, Andrade JS Jr, Havlin S, Herrmann HJ. Mitigation of malicious attacks on networks. Proceedings of the National Academy of Sciences. 2011;108(10):3838–3841. doi: 10.1073/pnas.1009440108 21368159 PMC3053993

[48] Liu X, Sun S, Wang J, Xia C. Onion structure optimizes attack robustness of interdependent networks. Physica A: Statistical Mechanics and its Applications. 2019;535:122374. doi: 10.1016/j.physa.2019.122374

[49] Khan MA, Javaid N. Computationally efficient topology optimization of scale-free IoT networks. Computer Communications. 2022;185:1–12. doi: 10.1016/j.comcom.2021.12.013

[50] Zhang C, Lei Y, Shen X, Li Q, Yao H, Cheng D, et al. Fragility Induced by Interdependency of Complex Networks and Their Higher-Order Networks. Entropy. 2022;25(1):22. doi: 10.3390/e25010022 36673163 PMC9858052

[51] Rossi R, Ahmed N. The network data repository with interactive graph analytics and visualization; 2015.

